# Deficits in medial prefrontal cortex parvalbumin expression and distraction-dependent memory in rats and mice in the sub-chronic phencyclidine model for schizophrenia

**DOI:** 10.3389/fncel.2025.1669050

**Published:** 2025-10-29

**Authors:** Katie R. Landreth, Jacob Juty, Neveen Mansour, Patricia Radu, Jennifer Fletcher, Imane Benalla, Ben Grayson, Rasmus S. Petersen, Michael K. Harte, John Gigg

**Affiliations:** 1 Division of Pharmacy and Optometry, The University of Manchester, Manchester, United Kingdom; 2 Division of Neuroscience, The University of Manchester, Manchester, United Kingdom

**Keywords:** schizophrenia, phencyclidine, pre-clinical model, parvalbumin, object memory, social cognition, distraction, proactive interference

## Abstract

**Introduction:**

Cognitive impairments associated with schizophrenia (CIAS) include deficits in declarative memory. This is associated with an inability to maintain information in short-term memory when distracted, and increased sensitivity to proactive interference. These CIAS may partly result from decreased expression of parvalbumin (PV) in medial prefrontal cortex (mPFC) interneurons. The sub-chronic phencyclidine (scPCP) rodent is a widely used model for schizophrenia that recapitulates CIAS, including declarative memory, social cognition and mPFC PV deficits. Thus, distraction before the test phase in novel object recognition (NOR) produces robust declarative memory deficits in scPCP rats. Controlling for distraction in the single trial or continuous NOR paradigm (cNOR) protects memory recall, and multi-trial cNOR reveals increased sensitivity to proactive interference for object memory. Here, we sought to expand scPCP model cross-species validity by comparing these NOR/cNOR deficits across scPCP rats and mice. We then aimed to determine whether distraction-dependent deficits are conserved across object and social memory domains in scPCP mice, assessing sociability and social memory using automated mouse tracking to sub-classify social interaction behaviors.

**Methods:**

scPCP mice underwent cNOR testing over 11 trials, and the density of cellular PV expression in putative interneurons (PVIs) in the mPFC was determined. scPCP mice were additionally tested in the Three-Chamber Social Interaction (TCSI) task, investigating social preference and the sensitivity of social memory to distraction. Mouse movement was tracked with a deep-learning tool (DeepLabCut) to classify sniffing and rearing in the TCSI task.

**Result:**

Distraction-dependent NOR deficits were conserved across scPCP rats and mice, while the effects of proactive interference on cNOR testing were species-specific. TCSI testing showed that scPCP mice expressed diminished sociability overall and increased susceptibility to distraction for social memory, particularly for rearing behavior. There was a significant reduction in PVI density in the scPCP mouse mPFC.

**Discussion:**

These results extend the cross-species validity of the scPCP model in rodents. scPCP-induced susceptibility to distraction in mice is broadly comparable to that observed in scPCP rats and is conserved across object and social memory domains. These behavioral effects correlate with scPCP-induced decreases in PV expression in both species, further implicating altered mPFC excitatory-inhibitory balance in CIAS induction.

## 1 Introduction

Normal learning and memory requires intact memory acquisition, consolidation and retrieval processes, during which the memory must be protected from influence by extraneous distractors, and resistant to contagion from other recent, similar memories (proactive interference). Patients with schizophrenia show increased susceptibility to both distraction and proactive interference (Park et al., 2003; Kaller et al., 2014; Girard et al., 2018; Becske et al., 2022), indicating an impaired ability to sustain or direct attention to appropriate stimuli while inhibiting redundant memories, hindering day-to-day functionality (Velligan et al., 2000).

Maintaining and retrieving memories despite the presence of distractions or interference from similar memories requires appropriate attentional focus in a process that involves the prefrontal cortex (PFC), an area with known parvalbumin (PV) impairments in patients with schizophrenia (Beasley and Reynolds, 1997; Kaar et al., 2019). The integrative theory of PFC function (Miller and Cohen, 2001) posits that the PFC provides active support and maintenance during memory consolidation in the hippocampus during task delay, which is crucial for higher-level cognitive control such as resisting distraction and retrieving only relevant memories, with prelimbic (PL) and infralimbic (IL) subregions playing key roles in memory consolidation and recall (Euston et al., 2012), and the anterior cingulate cortex providing additional support for social cognition (Bush et al., 2000; Amodio and Frith, 2006). Evidence from patients with prefrontal damage substantiates this executive role of the PFC, with severe impairments only appearing under distraction and interference conditions (Shimamura et al., 1995).

To understand the role of the PFC in Cognitive Impairments Associated with Schizophrenia (CIAS), detailed investigations of behavioral deficits alongside the neural mechanisms underlying these impairments must be better described in validated animal models. This could be achieved preclinically using the sub-chronic phencyclidine (scPCP) rodent model for schizophrenia (Neill et al., 2010; Castañé et al., 2015; Cadinu et al., 2018; Li et al., 2024), where administration of the NMDA receptor antagonist phencyclidine (PCP) induces robust cognitive and molecular changes consistent with those observed in patients. In particular, scPCP-induced changes to the expression of PV or the density of parvalbumin-expressing GABAergic interneurons (PVIs) in the PFC and hippocampus are implicated in CIAS due to the importance of PVIs in maintaining normal gamma oscillations and synaptic excitatory/inhibitory balance within and between brain regions (van Bokhoven et al., 2018). Reduced PVI density has been previously reported in the scPCP rat PFC (McKibben et al., 2010; Amitai et al., 2012; Redrobe et al., 2012; Landreth et al., 2021) and hippocampus (Abdul-Monim et al., 2007; Jenkins et al., 2010), as well as male scPCP mouse PFC and hippocampal CA1 subregion (Shirai et al., 2015).

The susceptibility of memory to distraction in the scPCP model has been investigated using the Novel Object Recognition (NOR) task, which is considered to be equivalent to tasks of declarative memory in humans (Winters et al., 2010; Neugebauer et al., 2016). scPCP rats were found to be amnesic only when distracted after memory acquisition by handling and removal from the arena during the inter-trial interval (Grayson et al., 2014; Landreth et al., 2021). This was also observed in scPCP mice using an adapted NOR paradigm (Gigg et al., 2020). The continuous NOR (cNOR) task consists of multiple NOR trials conducted sequentially without the need for handling between trials, allowing for the effect of proactive interference to be probed in the absence of distraction. Our previous work (Landreth et al., 2021) showed increased susceptibility to proactive interference in scPCP rats over an 11-trial cNOR task, alongside reduced PVI density in the mPFC. Unlike neurodevelopmental models for schizophrenia such as maternal immune activation (Simanaviciute et al., 2024), whisking behaviors are not abnormal in scPCP rats during object exploration (Landreth et al., 2021), indicating that CIAS are a result of aberrant memory consolidation or retrieval processes, rather than impaired sensorimotor integration during memory acquisition.

While the effect of distraction on object memory maintenance over a delay has been established in the scPCP model, its role in other cognitive domains, such as social cognition, have not been probed directly. The Three-Chamber Social Interaction (TCSI) task, developed by Nadler et al. (2004) to assess social withdrawal in models for autism, enables investigation into both social preference and social memory while ensuring that all instances of social interaction are initiated by the test mouse. The TCSI task has been used previously with the standard, continuous (distraction-free) protocol in scPCP mice and rats (Brigman et al., 2009; McKibben et al., 2014), though this did not result in a scPCP-induced social memory deficit in either species. However, alternate social interaction testing methods (such as the dyad social interaction task) have revealed scPCP-induced reductions in social behaviors and increased avoidance of the conspecific rat (Snigdha and Neill, 2008a,b), suggesting the presence of an asocial phenotype in the scPCP rat model.

Here, we investigated whether introducing a distraction step is sufficient to induce amnesia for social stimuli in scPCP mice using the TCSI paradigm. Following DeepLabCut (Mathis et al., 2018) tracking of mouse movement, a novel automated classification method was applied to identify sniffing and rearing behaviors during the task, to determine whether scPCP-induction alters how mice interact with social stimuli (naive conspecific mice). We also applied the cNOR task to the scPCP mouse model for the first time in order to further characterize scPCP-behavioral deficits in mice, and these animals were assessed by immunohistochemical analysis of PVI density in mPFC subregions (ACC, PL and IL). We present standard and continuous NOR data alongside our equivalent data from scPCP rats in order to increase cross-species validation of these behavioral paradigms in the scPCP model.

## 2 Materials and methods

### 2.1 Animals

Forty-eight adult female C57BL/6J mice (Charles River, UK) weighing 19.84 g ± 1.23 g (mean ± sd) at the beginning of testing were housed in groups of four in Techniplast ventilated cages at 20 °C ± 2 °C and humidity 55% ± 5%, maintained on a 12:12 h light:dark cycle with lights on at 07:00 h (Biological Services Facility, University of Manchester). Mice had *ad libitum* access to standard rodent chow (Special Diets Services, UK) and water. All procedures were performed under Home Office UK project licenses in accordance with the Animals (Scientific Procedures) Act UK 1986 and approved by the University of Manchester Animal Welfare and Ethical Review Body.

### 2.2 Sub-chronic phencyclidine administration

Mice were injected with 10 mg/kg phencyclidine hydrochloride (PCP HCl: 2 mg/mL in 0.9% saline; the scPCP group) or 0.9% saline (the scVehicle group) subcutaneously (5 mL/kg) once daily for 10 consecutive days (Gigg et al., 2020) followed by a washout period of 7 days prior to behavioral testing (including any test habituation), during which minimal handling occurred.

**Figure 1:**
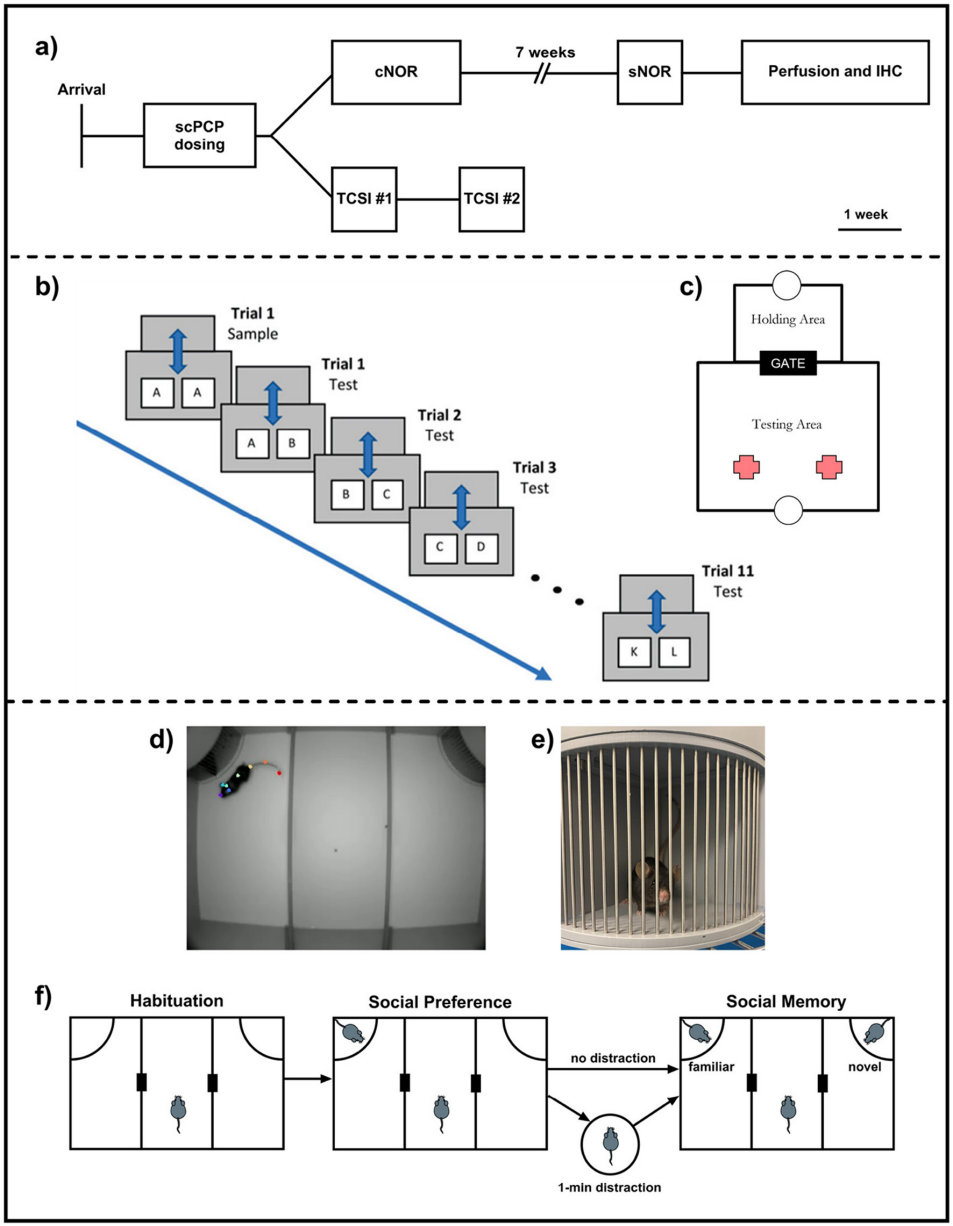
**(a)** Experimental design and study timeline; **(b)** Continuous NOR (cNOR) apparatus and **(c)** cNOR trial design; **(d)** Three-Chamber Social Interaction (TCSI) apparatus with a test mouse tracked using eight anatomical landmarks, **(e)** a conspecific mouse in the TCSI cage. Bars allow for limited contact when initiated by the test mouse. **(f)** TCSI trial design with both “distraction” and “no distraction” task conditions.

After scPCP administration, cage groups were assigned randomly to “object memory” or “social memory” sub-cohorts (see Figure 1a for experimental design and timeline). The “object memory” sub-cohort (*n* = 12/group) underwent continuous NOR (cNOR) and standard NOR (sNOR) testing, and brains were collected for immunohistochemical analyses. The “social memory” sub-cohort (*n* = 12/group) was tested in the Three Chamber Social Interaction (TCSI) task.

### 2.3 Continuous novel object recognition

#### 2.3.1 Apparatus

The cNOR testing apparatus (Campden Instruments Ltd., UK), consisted of two chambers, each with a food tray into which liquid food reward (Yazoo Strawberry milkshake; FrieslandCampina UK, Horsham, UK) could be dispensed. The chambers were separated by a gate, and an overhead camera recorded object exploration by the test mouse. The food dispensers, gate and camera were managed by ABET II software (Campden Instruments Ltd, UK).

#### 2.3.2 Habituation

Mice were habituated to the arena and trained to shuttle between the testing and holding areas as described in Chan et al. (2018) and as previously used by us (Landreth et al., 2021). Mice were mildly food restricted (2.8 g/mouse/day) from day one of habituation until the end of cNOR testing.

#### 2.3.3 Testing

cNOR testing was carried out as described previously in Landreth et al. (2021). Mice shuttled to the testing area for a 2-minute acquisition phase, where they explored two identical objects (A+A). After 2 min, the gate re-opened, the reward was dispensed into the holding area tray, prompting the mouse to shuttle to this area for a 1-minute ITI, during which the objects were swapped for a clean copy of object A and a novel object B. After the ITI, the gate re-opened, the reward was dispensed into the testing area tray, and the mouse re-entered the testing area for a 2-minute exploration period. This process was repeated a total of 12 times, with one acquisition trial (objects A+A) and 11 retention trials (A+B, B+C, C+D,...K+L; Figures 1b, c).

#### 2.3.4 Standard novel object testing in the continuous novel object recognition apparatus

Seven weeks after the end of cNOR testing, mice underwent an additional NOR task in the cNOR apparatus. Mice were placed into the testing area by the experimenter for a three minute acquisition phase, then removed and placed into an unfamiliar holding arena for one minute, before being placed back into the testing area for a three-minute retention phase. Three-minute task phases were chosen to match equivalent data in Landreth et al. (2021), and a distinct set of objects was used for this task.

#### 2.3.5 Scoring cNOR and sNOR behaviors

All trials were recorded via an overhead camera and analyzed using a Novel Object Recognition Task Timer (https://jackrrivers.com/program/). Exploration was defined as actively sniffing, licking or biting the object, and these exploration times were used to calculate a discrimination index (DI):


$$DI=\left(Time_{\text{novel}}-Time_{\text{familiar}}\right)/Time_{\text{total}}$$

A more positive DI value indicates a preference for novelty, while a DI of zero indicates no preference. Performance in the cNOR task over multiple trials was also assessed with a cumulative DI (cDI), the mean of the DIs from the current and prior trials.

### 2.4 Three-chamber social interaction

#### 2.4.1 Apparatus

The Three-Chamber Social Interaction (TCSI) apparatus (O'Hara, Japan) consisted of a large box divided into three equal sections with small gaps to allow the test mouse to move freely between the three chambers. Mouse movements during the task were recorded using an overhead camera for downstream analysis. Removable cages in the top left and right corners of the apparatus allowed conspecific mice to be placed into the TCSI box for exploration by the test mouse (Figures 1d, e).

#### 2.4.2 Habituation

Mice were habituated to the TCSI apparatus with two empty cages for 10 min. Each test mouse was placed individually into the center third of the box and allowed to explore freely.

#### 2.4.3 Social preference

Immediately following the habituation phase, one cage was replaced with an identical cage containing a novel conspecific mouse, while the other cage remained empty. The test mouse then freely explored for a further 10 min.

#### 2.4.4 Social memory

After completion of the social preference phase, the remaining empty cage was replaced with one containing a novel conspecific mouse, while the test mouse experienced one of two conditions (Figure 1f): **No Distraction**—the test mouse entered the middle section of the TCSI box while cages were swapped. **Distraction**—the mouse was removed from the TCSI apparatus and placed into an unfamiliar holding box for a one-minute inter-trial interval while the cages were swapped.

In both task conditions, the previously novel mouse introduced during the social preference phase then became the familiar mouse for the subsequent 10-minute social memory task phase. The position of the novel and familiar mice were counterbalanced across treatment groups and task conditions.

#### 2.4.5 Video analysis

Mouse movement tracking during the TCSI task was conducted using DeepLabCut (DLC v3.0.0rc8), a markerless pose estimation tool based on deep learning (Mathis et al., 2018; Gantar et al., 2025). We manually labeled eight anatomical landmarks on the mouse: the snout, left ear, right ear, neck point, mid-body point, tail base, mid-tail point, and tail end. A neural network was trained using a ResNet-50 backbone, allowing for accurate 2D tracking of the mouse's posture and location throughout the task. In addition to labeling the animal, reference points within the TCSI apparatus were also annotated to facilitate spatial analyses. This enabled precise mapping of mouse trajectories and quantification of time spent near each chamber, as well as classification of specific behaviors (sniffing and rearing) expressed in proximity to each cage.

The location of each body landmark, for each frame, was extracted from DeepLabCut tracking data. Only frames where the mid-body point was detected with a confidence score ≥ 0.6 were included. Interaction time was defined as the duration during which the snout point of the test mouse was within 5 cm of either conspecific cage. If the snout was not confidently tracked, the mid-body point was used instead. Interaction time was computed by summing qualifying frames and converting to seconds (25 fps).

Sniffing and rearing behaviors were automatically classified from DeepLabCut tracking data based on the positions of the snout and mid-body points. Sniffing was quantified as instances where the mouse's snout was located near the lower section of the cage, a region typically associated with close-range investigation of social or physical cues through the cage bars. Although this head positioning was not exclusively indicative of sniffing, it was consistently associated with the behavior. This association was verified through visual inspection of the videos, and such events were therefore classified as sniffing.

Rearing was associated with the mouse's snout being located in the upper section of the cage, which typically reflects the animal elevating its forelimbs against the cage wall. When the snout was not visible, often due to it moving out of the camera's field of view, rearing was inferred based on the position of the mid-body point. This classification was supported by visual inspection of the videos. Behavioral labels were smoothed using a 5-frame (200 ms) moving average to reduce frame-to-frame variability. An example video showing anatomical landmarks and behavioral labels can be found in Supplementary Figure 1.

#### 2.4.6 Scoring TCSI behaviors

Discrimination index (DI) values were calculated for social preference and social novelty, as in Section 2.3.5, with a positive DI indicating a preference for social interaction (in the social preference phase) or novelty (in the social memory phase). To assess preference over time, the running total of the DI was calculated per minute throughout each trial by summing total exploration of each cage up to that point.

### 2.5 Immunohistochemistry

#### 2.5.1 Tissue collection and processing

Mice were anesthetized with inhaled isofluorane and perfused transcardially, first with 1x phosphate-buffered saline (PBS) and then with 4% paraformaldehyde (PFA). Brains were removed and stored in 4% PFA overnight, then dehydrated in 30% sucrose in dH_2_O before flash-freezing. Frozen tissue was sectioned coronally to 30μm using a cryostat (Leica Biosystems, UK) and stored in a cryoprotectant solution (30% ethylene glycol, 30% glycerol, 10% PBS and 30% dH_2_O) at -20 °C. Medial prefrontal cortex (mPFC) subregions (anterior cingulate cortex, prelimbic and infralimbic cortices) were then delineated using the Allen Mouse Brain Atlas (mouse.brain-map.org/static/atlas), and stained for parvalbumin.

#### 2.5.2 Parvalbumin staining

Sections were washed three times in PBS (5 min/wash) then bathed in heated citrate buffer at 80 °C for 30 min. After three further PBS washes, sections were treated with hydrogen peroxide (88.1% PBS, 10% methanol, 1.5% H_2_O_2_, 0.4% Triton x-100) for 30 min, washed twice in PBS for 5 min each, transferred into protein block (94.6% PBS, 5% normal horse serum, 0.4% Triton x-100) and then incubated in PV primary antibody (1:2,000; Swant, Switzerland) diluted in protein block overnight at 4 °C. Samples were washed twice in PBS for 5 min each and incubated for 2h with anti-mouse secondary antibody (1:200; Vector Laboratories, UK; diluted in protein block), washed twice in PBS for 5 min each and then transferred to Vectastain ABC solution (Vector Laboratories) for 45 min. Samples were again washed twice in PBS for 5 min each before being stained with DAB substrate kit (Vector Laboratories; with or without Nickel) until sufficient staining was seen, washed in distilled water, mounted on slides and left to dry overnight. Finally, samples on slides were dehydrated for 5 mins each in 70%, 90% and then 100% ethanol, followed by Histoclear for 5 mins. When dry, samples were mounted with DPX (Sigma-Aldrich, UK).

#### 2.5.3 Determining PVI density

**Figure 2:**
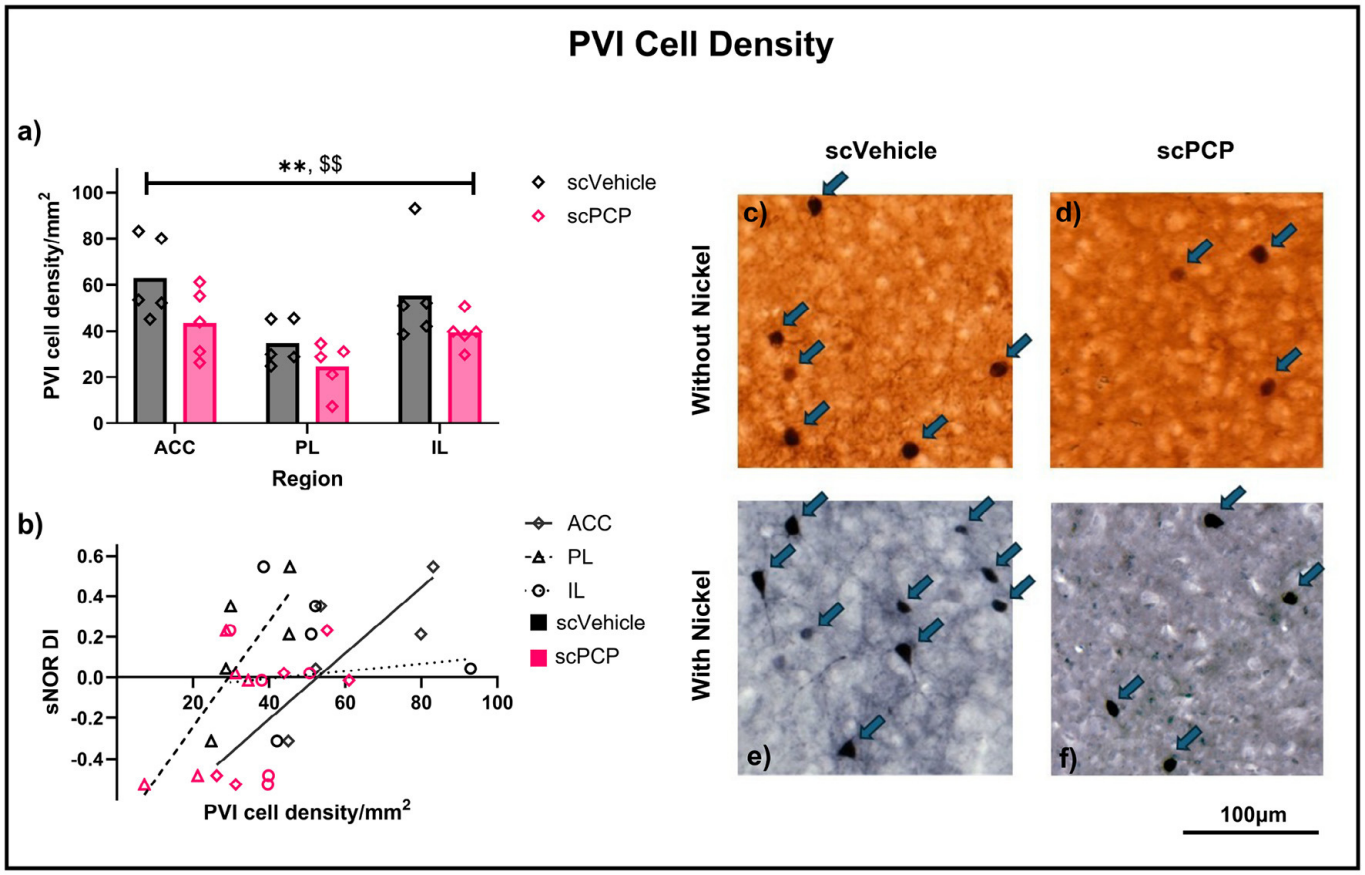
**(a)** PVI density in the anterior cingulate cortex (ACC), prelimbic (PL) and infralimbic (IL) areas of the mPFC of scVehicle and scPCP mice. Data were compared using a two-way ANOVA with *post-hoc* Sidak's comparisons; **(b)** Pearson's r correlations describing the relationship between standard NOR performance and density of PVIs in the mPFC subregions; **(c–f)** Typical examples of parvalbumin staining from mouse mPFC without **(c, d)** or with **(e, f)** nickel. *n* = 5/group, **p < 0.01 main effect of treatment, ^$$^*p* < 0.01 main effect of region.

Slides were imaged using the University of Manchester Bioimaging SlideScanner service and viewed using CaseViewer (3D-Histech). Regions of interest (ACC, PL and IL) were delineated manually, guided by the Allen Mouse Brain Atlas (mouse.brain-map.org/static/atlas), and PVIs were counted by experimenters blinded to treatment condition. PVI counts for a subset of sections were moderated by a second blinded experimenter. See (Figures 2c–f) for typical examples of parvalbumin staining.

### 2.6 Statistical analysis

PVI cell density was compared across treatment groups and regions using two-way ANOVA with *post-hoc* Sidak's multiple comparisons tests. Behavioral data were analyzed by either two-way repeated measures ANOVA or mixed measured analyses, followed by *post-hoc* Sidak's multiple comparisons. DIs from minutes 1–2 of the TCSI social memory task phase were also compared to an expected value of zero using one-sample t-tests. The relationship between sNOR DIs and mPFC PVI density was assessed using a Pearson's r correlation. All analyses were performed using GraphPad Prism (v10.4).

## 3 Results

### 3.1 Novel object recognition

One mouse did not complete cNOR training and was excluded from these analyses.

#### 3.1.1 Single trials

**Figure 3:**
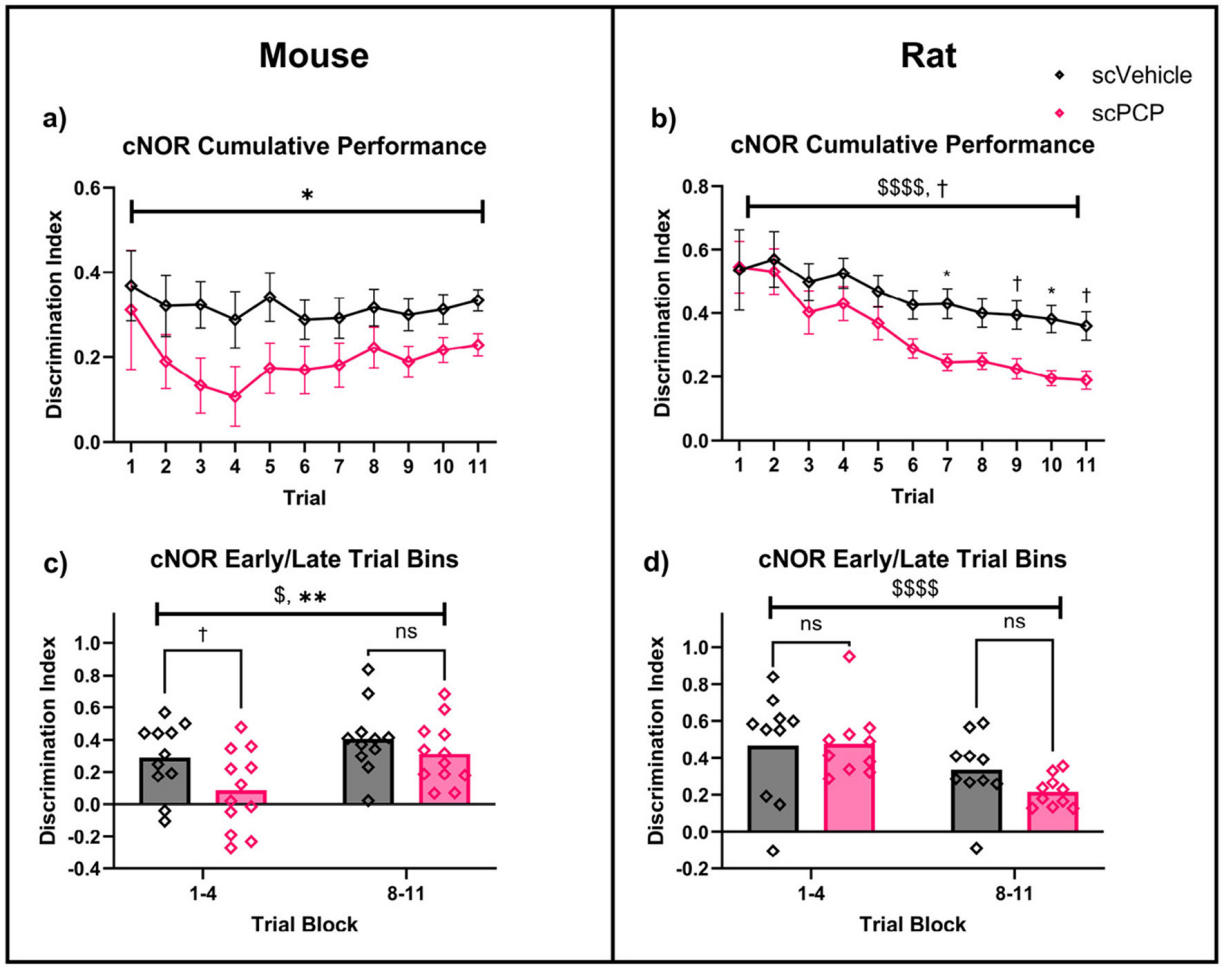
Two-way repeated measures ANOVA with *post-hoc* Sidak's multiple comparisons for scVehicle and scPCP mice **(a, c)** and rats **(b, d)** in trial one of the continuous NOR (cNOR) and standard NOR (sNOR) task. Mice **(a)**, and rats **(b)** both showed intact performance in trial one of the cNOR (distraction-free) task. scPCP mice **(c)**, and scPCP rats **(d)** were impaired in the sNOR task (after being distracted during the inter-trial interval). *n* = 10–12/group, ^†^0.05 ≤ *p* ≤ 0.08 trending effect of object; ^$$^*p* < 0.01; ^$$$$^*p* < 0.0001 main effect of object; *p < 0.05, **p < 0.01, ****p < 0.0001 *post-hoc* group-wise comparisons. scPCP rat data taken from Landreth et al. (2021) with permissions for reuse under CCBY4.0 license.

Exploration of familiar and novel objects in trial one of the cNOR task and the sNOR task was assessed with two-way repeated measures ANOVA. In mice, there was a significant effect of object (F_1, 21_ = 12.89, *p* < 0.01) on exploration in trial one of the cNOR task (without distraction; Figure 3a). *Post-hoc* Sidak's comparisons found that both scVehicle (*p* < 0.05) and scPCP (*p* < 0.05) groups explored the novel object more than the familiar object. Introducing distraction by performing a standard NOR task in the cNOR apparatus also produced a trending effect of object (F_1, 21_ = 3.61, *p* = 0.071), as well as a significant object*treatment interaction (F_1, 21_=12.47, *p* < 0.01; Figure 3c). *Post-hoc* Sidak's multiple comparisons tests found that scVehicle mice explored the novel object significantly more than the familiar object during the sNOR task (*p* < 0.01), while the scPCP mice did not (*p* = 0.469).

Equivalent data taken from our previous work in scPCP rats (Landreth et al., 2021), also found a significant effect of object (F_1, 18_ = 41.92, *p* < 0.0001) on exploration in trial one of the cNOR task (Figure 3b), with no overall effect of treatment or treatment*object interaction. *Post-hoc* comparisons showed a significant novelty preference in scVehicle (*p* < 0.0001) and scPCP (*p* < 0.01) groups. In the sNOR task, there was a significant effect of object (F_1, 18_ = 10.53, *p* < 0.01) and object*treatment interaction (F_1, 18_ = 5.66, *p* < 0.05; Figure 3d), but not of treatment. *Post-hoc* Sidak's tests were significant for scVehicle (*p* < 0.01) but not scPCP (*p* = 0.796) rats in the sNOR task.

Two-way ANOVA also compared performance DIs across species. There was a significant effect of treatment (F_1, 39_ = 16.89, *p* < 0.001), but not of species, treatment*species interaction, or *post-hoc* group comparisons, on sNOR performance. cNOR trial one performance was not effected by treatment, species, or treatment*species interaction, and no significant *post-hoc* comparisons were found. Together, these data indicate that both scPCP rats and mice possess intact object memory when tested in a Novel Object Recognition task in the absence of distraction (cNOR trial one), but that scPCP rodents of both species are amnesic after being distracted during the inter-trial interval (the sNOR task).

#### 3.1.2 Continuous trials

**Figure 4:**
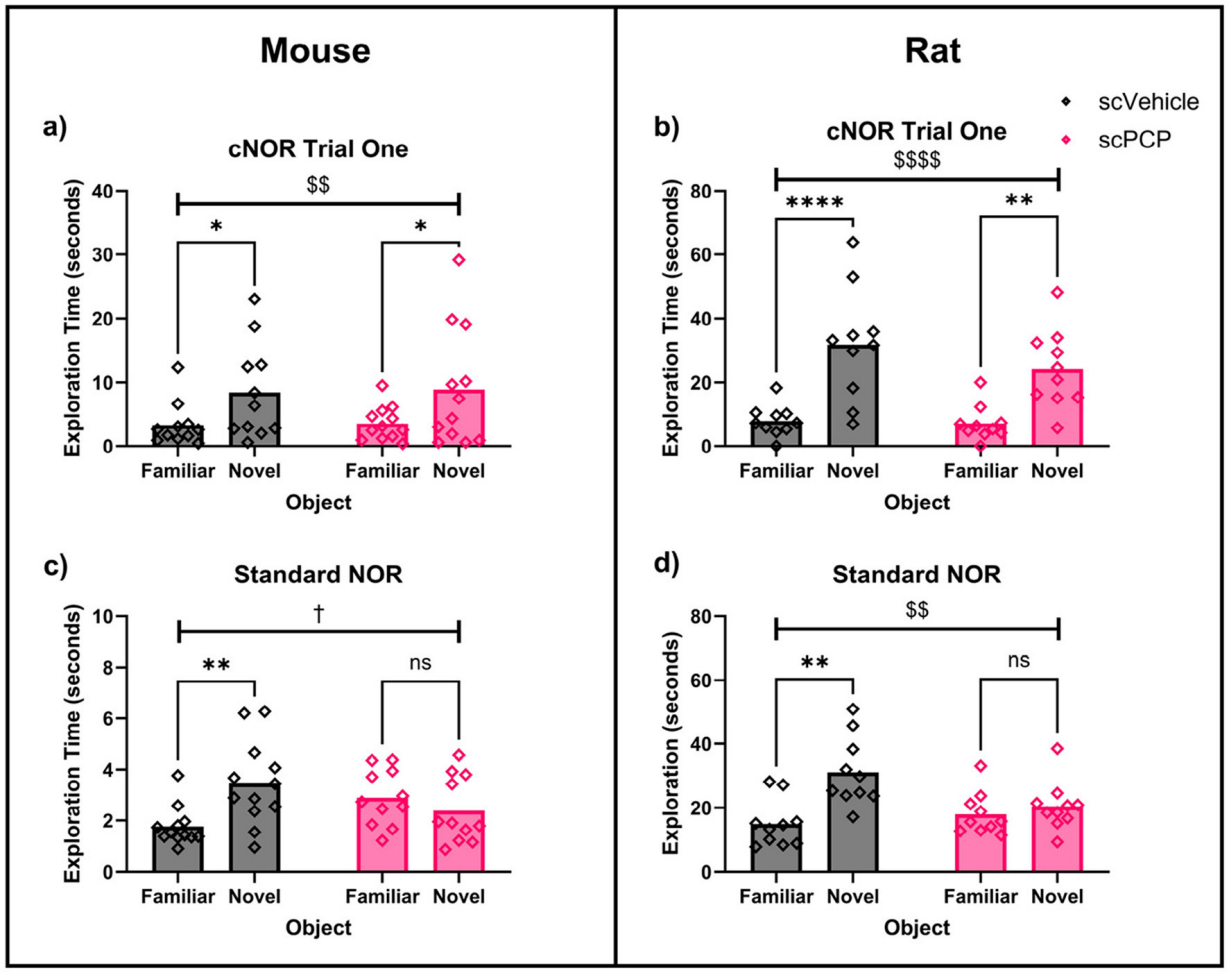
Performance of scPCP mice **(a, c)** and rats **(b, d)** over continuous NOR trials, analyzed using two-way repeated-measures ANOVA with *post-hoc* Sidak's multiple comparisons. **(a)** scPCP performance was significantly impaired overall, **(b)** scVehicle and scPCP rat performance worsened over time, particularly in the scPCP group, **(c)** mice performed worse in early trials compared to late trials, with scPCP mouse performance consistently lower than scVehicle, **(d)** in rats, overall performance was lower in the later trial block, irrespective of treatment. *n* = 10–12, ^$^*p* < 0.05, ^$$$$^*p* < 0.0001 main effect of trial; ^†^0.05 ≤ *p* ≤ 0.08 trending effect of treatment, *p < 0.05, **p < 0.01 main effect of treatment. scPCP rat data taken from Landreth et al. (2021) with permissions for reuse under CCBY4.0 license.

Cumulative performance in the cNOR task over 11 continuous trials was assessed using a two-way repeated measures ANOVA. In mice, there was a significant overall effect of treatment (F_1, 21_ = 4.562, *p* < 0.05), but not of trial number, or trial*treatment interaction, and no significant *post-hoc* comparisons (Figure 4a). Equivalent cumulative performance in rats (originally presented in Landreth et al., 2021) also showed a significant effect of trial (F_2.308, 39.23_ = 14.69, *p* < 0.0001) as well as a trending effect of treatment (F_1, 17_ = 3.724, *p* = 0.071), but not trial*treatment interaction (Figure 4b). *Post-hoc* Sidak's comparisons found a significant difference in rat group performance at trials 7 and 10 (both *p* < 0.05), and trending differences at trials 9 (*p* = 0.078) and 11 (*p* = 0.062).

Early and late task performance was assessed by averaging DIs for trials 1–4 and 8–11 respectively. In mice, a two-way repeated measures ANOVA found a significant effect of trial block (F_1, 21_ = 4.906, *p* < 0.05) and of treatment (F_1, 21_ = 9.410, *p* < 0.01), with no trial block*treatment interaction (Figure 4c). *Post-hoc* Sidak's comparisons found a trending treatment difference in early performance (*p* = 0.064), but not of late trial performance. Matched performance in rats showed a significant effect of trial block (F_1, 18_ = 29.39, **p** < 0.0001) but not of treatment or trial block*treatment interaction, and no significant *post-hoc* group comparisons were found (Figure 4d).

Early and late task performance DIs were also assessed separately across species. There was a significant effect of species (but not of treatment, or species*treatment interaction) on performance in trials 1-4 (F_1, 39_ = 15.24, *p* < 0.001). This species difference was not seen in trials 8–11, however, there was a trending overall effect of treatment on late task performance (F_1, 39_ = 3.775, *p* = 0.059). *Post-hoc* Sidak's comparisons of early task performance found that scPCP group performance differed across species (*p* < 0.001), but scVehicle performance did not, and no significant differences were identified in late task performance. In summary, cNOR performance in rats progressively worsened throughout the task, with scPCP rat performance declining at a faster rate than that of scVehicle control, such that an effect of scPCP treatment began to emerge from trial 7 onwards. scPCP mice did not follow this trend; an overall treatment effect was observed throughout the 11 cNOR trials, with the most pronounced differences appearing during the first four trials.

### 3.2 Three-chamber social interaction

#### 3.2.1 Social preference

**Figure 5:**
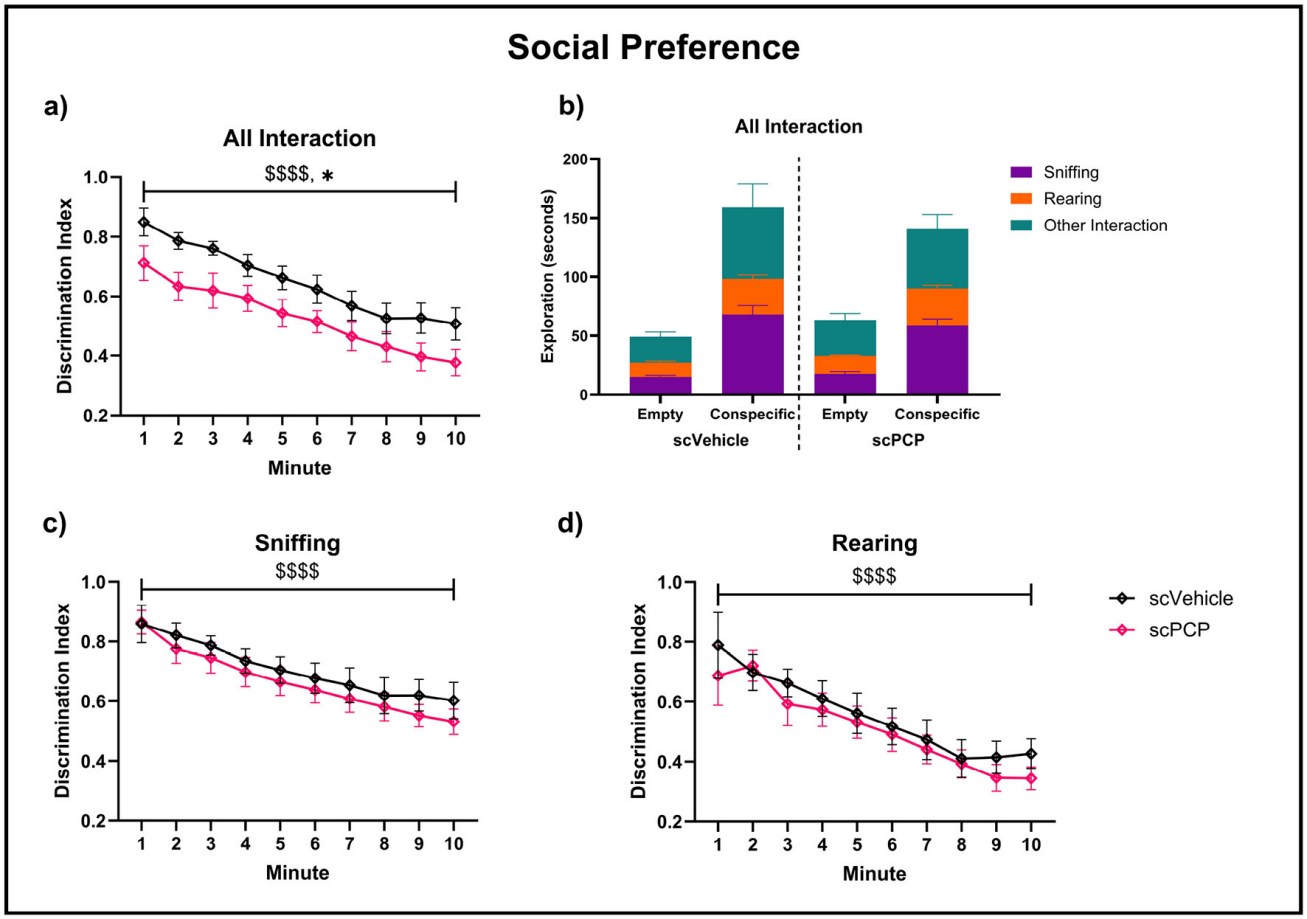
Pro-social exploratory behaviors throughout a 10-min three-chamber social interaction (TCSI) task phase, with plots **(a, c, d)** analyzed using two-way repeated measures ANOVA. Discrimination indexes (DIs) above 0 indicate preference for a cage containing a conspecific mouse over an empty cage for **(a)** total interaction time, **(b)** total interaction time subdivided to show sniffing, rearing and other interactions during this time, **(c)** sniffing and **(d)** rearing behaviors. *n* = 12/group, ^$$$$^*p* < 0.0001 main effect of minute, *p < 0.05 main effect of treatment.

Each mouse was tested twice in the TCSI task, with counterbalanced “distraction” and “no distraction” conditions occurring after the social preference phase, and task protocols being otherwise identical until this point (Figure 1f). Therefore, performance during the social preference task phase was averaged across both test repeats. There was a significant effect of scPCP treatment (F_1, 22_ = 6.041, *p* < 0.05) and time (F_2.004, 44.10_ = 27.50, *p* < 0.0001) on all interactions during the social preference task phase, as measured by DI (Figure 5a). All interactions were subdivided into sniffing, rearing, and other interactions (where the mouse was within 5cm of the cage, but not sniffing or rearing). The proportion of time performing each behavior is visualized in Figures 5b–d. Sniffing and rearing behaviors were significantly affected by time (sniffing F_1.691, 37.19_ = 23.38, *p* < 0.0001; rearing F_2.512, 55.26_ = 15.44, *p* < 0.0001) but not by scPCP treatment. No significant *post-hoc* differences were found in any of these measures.

#### 3.2.2 Social memory

**Figure 6:**
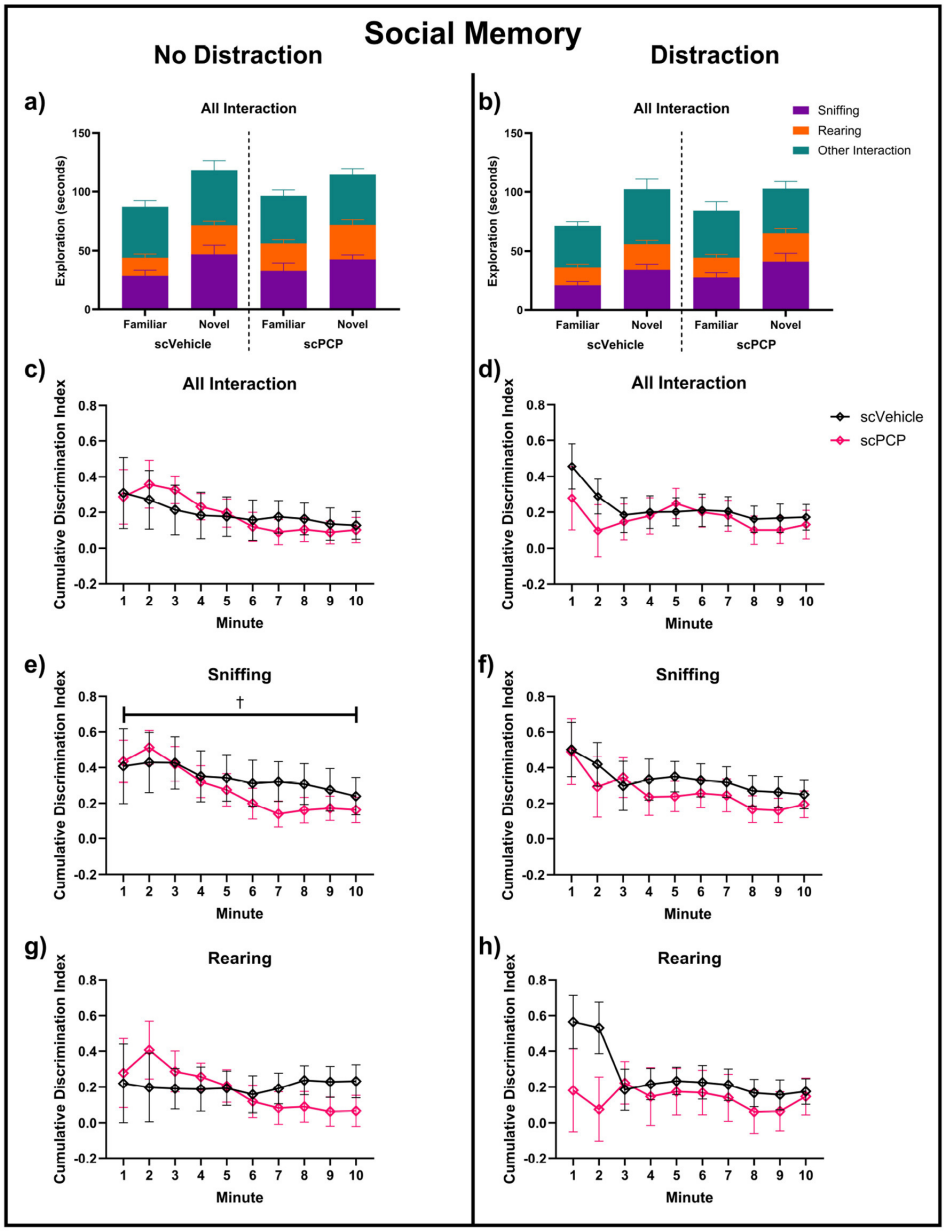
Exploration of cages containing novel and familiar conspecific mice across a 10-min task in no-distraction **(a, c, e, g)** and distraction **(b, d, f, h)** conditions; **(a, b)** total interaction time, sub-divided into time spent sniffing, rearing and other interactions during this time. Discrimination indexes (DIs) above 0 indicate preference for a cage containing a novel mouse for **(c, d)** total interaction, **(e, f)** sniffing, and **(g, h)** rearing behaviors, and are analyzed using two-way repeated measures ANOVA. *n* = 12/group, ^†^trending 0.05 ≤ *p* ≤ 0.08 main effect of minute.

Performance in the social memory task phase was probed separately for both the distraction and no-distraction conditions. Total interaction time, and the proportion of this time spent performing sniffing, rearing or other behaviors, are visualized in Figures 6a, b. Two-way repeated measures ANOVA found no effect of treatment or time on the time spent near the cages. When subdivided into sniffing and rearing behaviors for both task conditions, there was a trending effect of time (F_1.990, 43.78_ = 2.93, *p* = 0.064) on no-distraction sniffing DIs in this task phase, but no other effects of time or treatment were found across task conditions or exploratory behaviors (Figure 6). No significant *post-hoc* differences were found in any of these measures.

**Figure 7:**
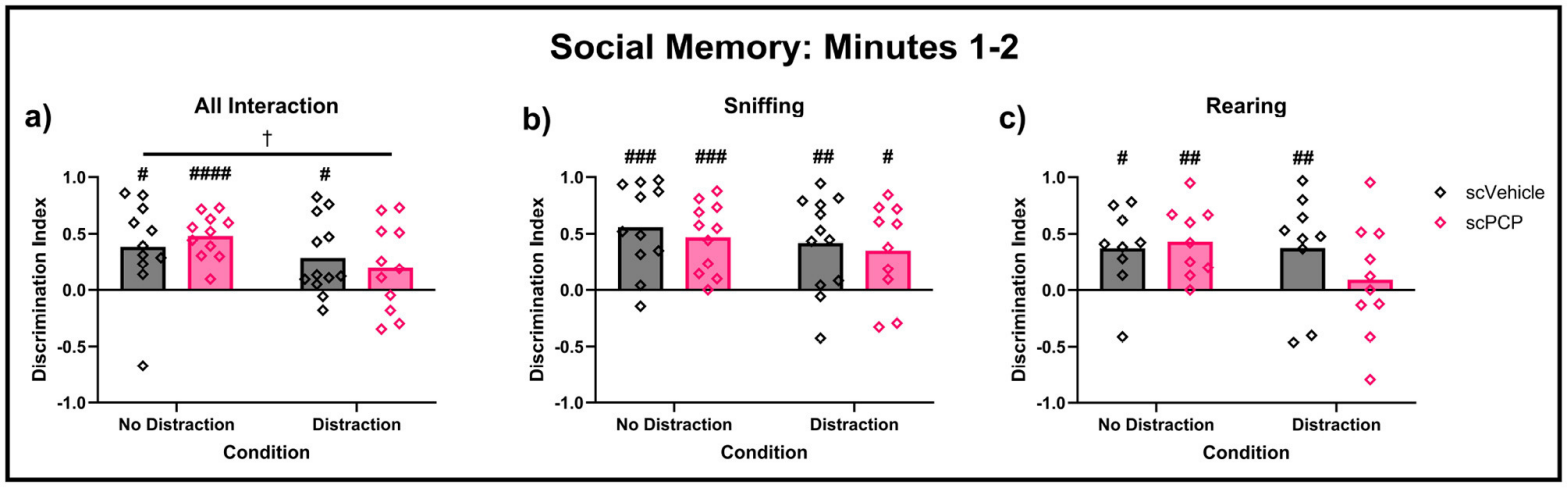
Performance in the first two minutes of the TCSI social memory task, with Discrimination Indexes (DIs) of +1 and -1 removed. DIs were compared using two-way repeated measures ANOVA, and each group DI was compared to zero using one-sample *t*-tests. DIs above 0 indicate preference for a cage containing a novel mouse during minutes 1–2 of the task for **(a)** total interaction, **(b)** sniffing, and **(c)** rearing behaviors. *n* = 9–12/group, ^†^0.05 ≤ *p* ≤ 0.08 trending effect of task condition; ^#^<0.05, ^*##*^<0.01, ^*###*^<0.001 difference from zero. ^*####*^<0.0001

While no effects of scPCP treatment were found on social interactions across the full 10-minute task phase, Figure 6 suggested some treatment differences may be present in the first two minutes of the task. Therefore, social memory performance in the first two minutes was further investigated, and is shown in Figure 7. Mice with DI values of +1 or -1 were excluded from these analyses as preference for either cage could not be established where only one cage was explored. Mixed effects analyses found a trending effect of task condition (F_1, 19_ = 4.128, *p* = 0.056) on total interaction (Figure 7a). No significant effect of treatment, condition, or treatment*condition interaction was found. One-sample t-tests compared group performances to zero, with DIs significantly greater than zero indicating a significant preference for the novel mouse, indicating intact social memory. scPCP mice showed a significant preference for the novel conspecific throughout the no-distraction condition (total interaction t_10_ = 8.18, *p* < 0.0001; sniffing t_10_ = 5.08, *p* < 0.001; rearing t_8_ = 4.17, *p* < 0.01). After the introduction of a distraction, scPCP mice showed significant preference for novelty as measured by sniffing (t_9_, *p* < 0.05), but not in total interaction or rearing behaviors. scVehicle mice showed significant novelty preference in all measures and in both task conditions [total interaction distraction t_11_ = 2.96, *p* < 0.05, no-distraction t_10_ = 2.99, **p** < 0.05; sniffing distraction t_11_ = 3.44, *p* < 0.01, no distraction t_10_ = 4.76, *p* < 0.001; rearing distraction t_10_ = 3.19, *p* < 0.01, no distraction t_8_ = 3.09, *p* < 0.05]. No significant *post-hoc* differences were found in any of these measures.

Overall, we have observed an effect of scPCP treatment on the proportion of time these mice spent interacting with a conspecific mouse over an empty cage, indicating diminished social preference in the model, although this difference could not be explained by changes to sniffing or rearing behaviors. scPCP-induced social memory impairments were most clearly observed during the first two minutes of this task phase, where scPCP mice were amnesic only after being distracted during the inter-trial interval.

### 3.3 Immunohistochemistry

Average PVI density per region was first calculated for each mouse separately for both staining types, and a three-way ANOVA found a significant effect of region (F_2, 18_ = 7.313, *p* < 0.01) and treatment (F_1, 9_ = 8.154, *p* < 0.05), but not of staining type, or any interactions between these variables. Subsequently, data were combined across staining types and average PVI density was recalculated to include all sections for each mouse, regardless of staining type. Two-way ANOVA analyses on these data again found significant effects of region (F_2, 24_ = 7.069, *p* < 0.01) and treatment (F_1, 24_ = 8.055, *p* < 0.01), but no region*treatment interaction (Figure 2a). Treatment effects in each region were probed using *post-hoc* Sidak's multiple comparisons, and no significant differences were found, although scPCP mice had lower PVI density in each region (reductions of 30%, 29% and 28% in the ACC, PL, and IL, respectively). These data were also correlated to standard NOR performance, as measured by retention phase DI, and a significant positive relationship was found between sNOR DI and PVI density in the ACC (Pearson r = 0.8429, *p* < 0.01) and PL (Pearson r = 0.8192, *p* < 0.01) regions, but not the IL region (Figure 2b). Linear regression analyses for each of these relationships are reported in Table 1.

**Table 1:** Results of linear regressions modeling the relationship between standard NOR performance and density of PVIs in three regions of the mPFC.

**Region**	**R^2^**	**Slope**	**Y-intercept**	***p*-value**
ACC	0.710	0.016	-0.858	< 0.01
PL	0.671	0.026	-0.767	< 0.01
IL	0.008	0.007	0.359	0.803

## 4 Discussion

We first aimed to assess the effects of distraction (defined here as removal to a holding cage at the end of the acquisition phase followed by reintroduction to the arena at the end of the inter-trial interval) and proactive interference on performance in the cNOR task in scPCP mice, and compare this to previously reported behavior in scPCP rats. A scPCP-induced impairment in object recognition memory was observed in the standard NOR task, where mice were distracted during the inter-trial interval, but not when this distraction was removed in the first trial of the cNOR task. This is consistent with our equivalent data in rats (Landreth et al., 2021), with cross-species comparisons finding an overall effect of treatment in sNOR performance, but not in cNOR trial one, and no effect of species in either measure. In addition to other literature (Grayson et al., 2014; Gigg et al., 2020), this suggests that, in the scPCP model, susceptibility to distraction during the NOR task is conserved across species and is insensitive to NOR assessment method.

Performance across multiple trials in the cNOR task was species-specific. Previous work (Landreth et al., 2021) showed a gradual decrease in cNOR performance in rats over time as a result of proactive interference, and that scPCP rats appear to be more susceptible to these effects. However, we did not find an effect of trial on cNOR performance in mice in the present study. scVehicle cDIs across 11 cNOR trials appear relatively steady in mice, with a consistent preference for novelty that mirrors existing literature (Chan et al., 2018). scPCP group performance in mice diverges from scVehicle over the first four cNOR trials before rebounding and plateauing at a lower cDI, though a preference for novelty still remains. These differences are reflected in our cross-species statistical comparisons, with a significant effect of species being identified in early (but not late) task performance, as well as a trending treatment effect emerging in the late trial block. The performance of scVehicle and scPCP mice over trials 1–4 may represent an increased susceptibility to proactive interference after scPCP treatment similar to that of rats, albeit with an accelerated timeline. The lack of change in cDIs for the remainder of the task may then be due to a smaller working memory capacity in mice. Proactive interference necessitates continued existence of earlier, similar memories, so extinction of these memories during later trials would prevent the worsening of cNOR performance as a result of proactive interference. Future work aiming to rescue scPCP-induced susceptibility to proactive interference using therapeutic interventions may benefit from the accelerated timeline observed in mice, as only four cNOR trials would need to be conducted, meaning a higher throughput with behavior being tested over a shorter post-dosing window. Conversely, the more gradual separation of scVehicle and scPCP performance in rats may allow for more subtle behavioral changes to be observed after a therapeutic intervention. These factors should be considered when planning future cNOR experiments.

We next aimed to characterize social preference and memory performance in scPCP mice using the TCSI task. Our results show an overall scPCP-induced impairment in social preference, as measured by the sum of all interactions. This is consistent with previous findings (Brigman et al., 2009), where scPCP mice did not show a significant preference for a social stimulus over an empty cage. However, our automated behavioral tracking tool allowed us to separate sniffing and rearing behaviors from other types of social interaction during the TCSI task. Our results show that the overall impairment in social preference is not accounted for by changes in sniffing and rearing exploratory behaviors and suggests that, while active exploration of cages is similar across treatment groups, scPCP mice spend relatively less time near the social stimulus compared to the non-social stimulus, when not actively exploring. The current study did not measure additional behaviors found within this period of “other interaction”, however, future work may seek to investigate if any other behaviors, e.g. head-shaking [associated with positive symptoms of schizophrenia (Li et al., 2024)] or auto-grooming [linked to hyper-arousal and behavioral perseveration in models for schizophrenia and other neurodevelopmental disorders (Kalueff et al., 2016)] are driving the results observed here.

Although no significant effects of time or scPCP treatment were found across the full 10-minute TCSI social memory task phase, the data presented in Figure 6 suggested there may be differences in task performance during the first two minutes that are not reflected in the overall analysis. Assessing the first two minutes of the social memory task in isolation elucidates a clear distraction-dependent effect of scPCP treatment. Without distraction, scPCP mice show significant preference for novelty in all measures, whereas these mice do not show preference for novelty when measured by all interactions or rearing behaviors after the introduction of a distraction. scVehicle mice display this novelty preference in all instances regardless of distraction. This treatment effect may be confined to early in the task phase as the mice gain familiarity with the “novel” mouse during exploration. This finding suggests that the TCSI social memory task phase can be shortened significantly, improving throughput while increasing sensitivity.

mPFC function is vital for resisting distraction and directing attentional focus during task delay, and PVI impairments in this region have been identified in patients with schizophrenia (Beasley and Reynolds, 1997; Kaar et al., 2019). The PL and IL subregions of the mPFC are suggested to be particularly important for memory consolidation and recall, with other prefrontal areas unable to compensate for mPFC loss during these processes, as is possible during memory acquisition (Euston et al., 2012). The ACC, while also active during cognitive tasks, plays an important role in social interaction (Bush et al., 2000; Amodio and Frith, 2006). PVI deficits in each of these regions are, therefore, likely to impair performance in behavioral tasks such as those presented here. We found that scPCP mice had a significantly lower density of PVIs across all regions of the mPFC than scVehicle control. PVI density in the ACC and PL, but not IL, were significantly, positively associated with standard NOR performance. This PVI density data was collected from the “object memory” sub-cohort, meaning that a direct relationship between ACC PVI density and performance during social tasks could not be probed, and this provides an avenue for potential future work. Our findings are consistent with the existing scPCP literature describing reductions in PVI density in the PFC in mice (Shirai et al., 2015) and rats (McKibben et al., 2010; Amitai et al., 2012; Redrobe et al., 2012; Landreth et al., 2021). Overall, these findings add valuable cross-species validation to the scPCP model, as well as increasing face validity for the scPCP model as a pre-clinical model for CIAS in patients, with comparable behavioral and molecular changes reported in the clinical literature (Beasley and Reynolds, 1997; Park et al., 2003; Kaller et al., 2014; Girard et al., 2018; Kaar et al., 2019; Becske et al., 2022). Consistent findings across mice, rats and patients suggest that these phenomena are a result of conserved underlying neural mechanisms of relevance to schizophrenia.

In addition to the mPFC, other regions, such as the perirhinal cortex (PRC), are implicated in the induction of impaired object memory during short-delay tasks such as those reported here (Winters et al., 2008; Warburton and Brown, 2015), although lesion study data suggest PRC involvement in tasks with ITI lengths of more than three minutes (Norman and Eacott, 2004). Nevertheless, reduced PRC volume has been found to correlate with poor performance in NOR with a one-minute ITI in scPCP rats (Doostdar et al., 2019). PRC PVI density in the scPCP model has not yet been reported. The current study focused on PVI disruptions in the mPFC due to the crucial supporting role played by this region during memory consolidation, and the necessity for intact mPFC function during distraction and interference conditions in memory tasks (Shimamura et al., 1995). The mPFC PVI deficits reported here may hint at disrupted PRC function in the model due to the anatomical connectivity between the two regions (Deacon et al., 1983) and the potential for regional disinhibition to disrupt processing within efferent regions (Bast et al., 2017), however, direct investigation of PV expression in the PRC, and the effect of this on PVI physiology, would greatly improve our understanding of the mechanisms by which distraction induces CIAS in the scPCP model, and this should be a direction for future study. Additional measures of PVI activity, and of supporting structures such as peri-neuronal nets (PNNs), should also be considered, as these would further contextualize the deficits reported here. Finally, it is worth considering the impact of acute stress (Nelissen et al., 2018; Page et al., 2019), handling (Landreth et al., 2023), and food restriction (de Oliveira et al., 2022; Nguyen et al., 2025) imposed on these mice during behavioral testing, both on the behavioral measures and PVI expression measured here. If scPCP-model induction leads to differing susceptibility to these factors, then the results reported here would reflect an accumulated interaction of these effects alongside the pharmacological response to scPCP administration.

To conclude, we have found that distraction is necessary to impair recognition memory in object and social domains in scPCP mice, with this distraction-dependent amnesia being consistent with object memory impairments observed in scPCP rats. scPCP mice demonstrated diminished social preference overall, although this was not explained by changes to active exploratory behaviors, and this evidence for relative asociality concurs with findings of impaired dyad social interaction in scPCP rats. Different working memory capacities in rats and mice may explain species-specific cNOR task performance, with practical implications that should be considered when designing future behavioral studies. Finally, we have presented evidence for mPFC PVI deficits that are predictive of standard NOR performance in mice and show qualitative agreement with both scPCP rat and patient data.

## Data Availability

The raw data supporting the conclusions of this article will be made available by the authors, without undue reservation.
