# Supplementary material for: Deficits in medial prefrontal cortex parvalbumin expression and distraction-dependent memory in rats and mice in the sub-chronic phencyclidine model for schizophrenia
Source: Front Cell Neurosci. 2025 Oct 29;19:1669050. doi: 10.3389/fncel.2025.1669050 (PMC12605401; doi:10.3389/fncel.2025.1669050)
Supplement: Supplementary file 1 [file Presentation_1.pptx]

## Slide 1
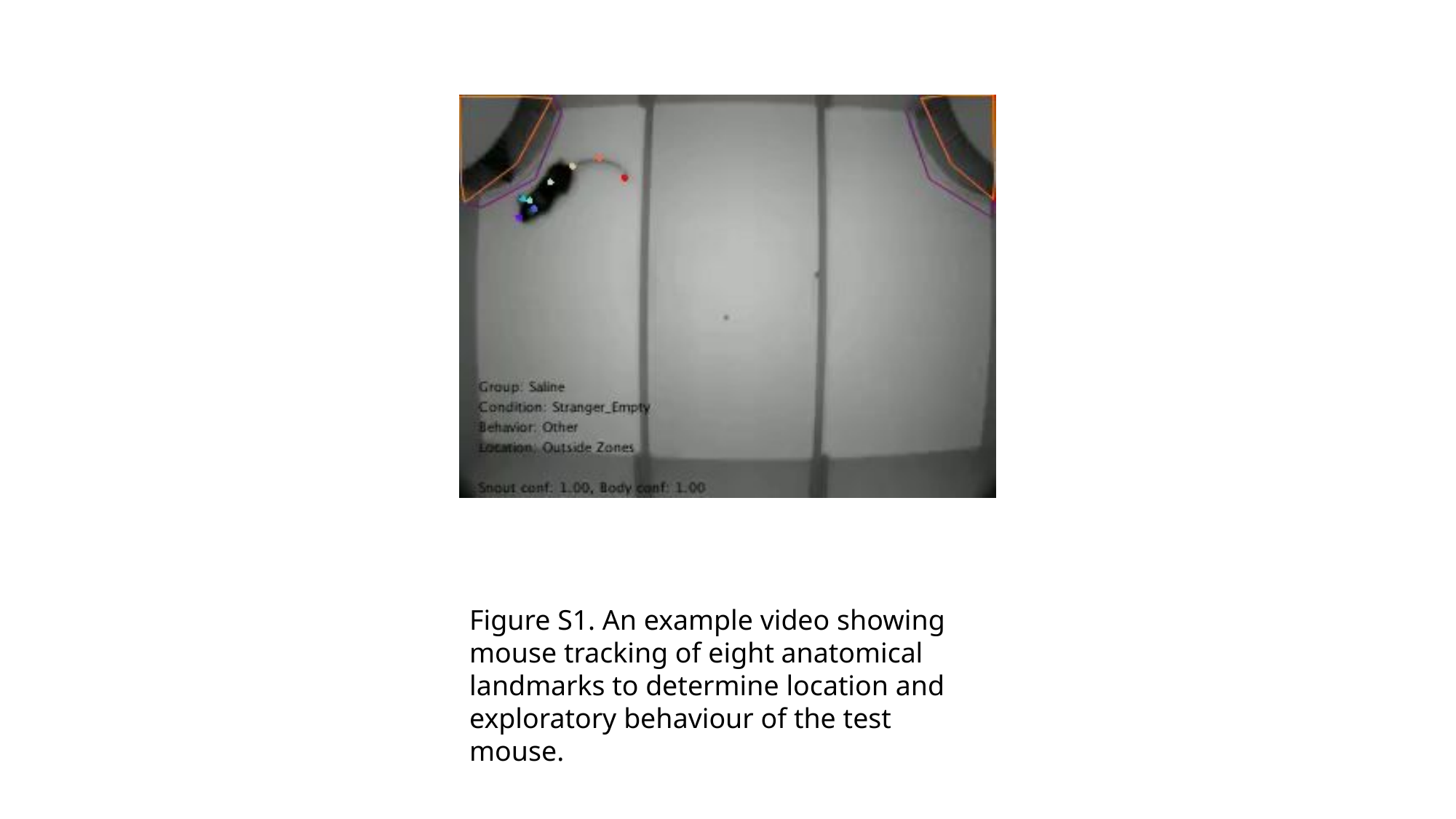

Figure S1. An example video showing mouse tracking of eight anatomical landmarks to determine location and exploratory behaviour of the test mouse.
